# Glucagon-like peptide-1 receptor agonists for obesity: Growing popularity met with growing questions over safety

**DOI:** 10.1371/journal.pmed.1004871

**Published:** 2026-01-14

**Authors:** Ariana M. Chao, Adam Gilden, Thomas A. Wadden

**Affiliations:** 1 Johns Hopkins University School of Nursing, Baltimore, Maryland, United States of America; 2 Division of General Internal Medicine, Johns Hopkins School of Medicine, Baltimore, Maryland, United States of America; 3 Division of General Internal Medicine, University of Colorado School of Medicine, Denver, Colorado, United States of America; 4 Department of Psychiatry, Perelman School of Medicine, University of Pennsylvania, Philadelphia, Pennsylvania, United States of America

## Abstract

In this Perspective, Ariana Chao, Adam Gilden and Thomas Wadden discuss why alongside the rising popularity of glucagon-like peptide 1 (GLP-1)-based medications for obesity, there are also concerns about side effects, long-term outcomes, and unregulated products.

## Teaser

Glucagon-like peptide-1 (GLP-1)-based medications, such as semaglutide and tirzepatide, have transformed obesity care. However, rising use brings concerns about side effects, long-term outcomes, and unregulated products. Ensuring safe access requires oversight, monitoring, and coordinated clinical care.

Originally developed for the management of type 2 diabetes, the glucagon-like peptide-1 (GLP-1) receptor agonist (RA), semaglutide, and GLP-1/glucose-dependent insulinotropic polypeptide dual RA, tirzepatide, have emerged as the most effective pharmacologic interventions for obesity treatment. Interest in these medications from healthcare professionals and the public has been unprecedented, with an estimated 700% increase in GLP-1-based prescriptions among people without diabetes from 2019 to 2023 [1]. This upsurge has been fueled by robust clinical trial efficacy data, favorable effects on metabolism and obesity-related complications, growth in recognition of obesity as a chronic medical disease, and pervasive media exposure. Prescribing has increased in both primary care and specialty settings, including by providers who had not previously treated obesity but have since integrated these medications into their practices. Yet the rapid expansion of use—which has extended beyond approved indications, populations included in clinical trials, and durations tested in most clinical trials—raises important questions regarding safety.

## Clinical trials may not fully capture the real-world risks of GLP-1-based medications

As with any medication, GLP-1-based medications have risks. The short- to medium-term safety profiles of semaglutide and tirzepatide have been well-characterized in over 40 phase-3 studies. There have also been numerous trials of GLP-1-based medications or combinations that are in the pipeline (e.g., cagrilintide/semaglutide, orforglipron, retatrutide, survodutide). Together, these studies have been conducted in a range of populations, including people with type 2 diabetes, cardiovascular disease, congestive heart failure, obstructive sleep apnea, and chronic kidney disease. Many trials have demonstrated comparable side effects, though prevalence rates have varied [2,3]. In phase 3 trials, the most common adverse events have been gastrointestinal, including nausea, vomiting, diarrhea, constipation, bloating, and abdominal pain [4]. These effects tend to be mild, are most pronounced during dose escalation, and occur in up to half of patients. Other common side effects have included dyspepsia, fatigue, and headache. Rare but serious side effects, including cholelithiasis, pancreatitis, and acute kidney injury are noted in the drugs’ prescribing information [2–4] and regulators are monitoring for such possible serious side effects [5].

Attention to side effects—particularly those that may be serious, rare, and not fully detectable in clinical trials—continues to warrant close scrutiny. Controlled trials often have strict inclusion/exclusion criteria and relatively short follow-up durations, which may not capture uncommon or delayed adverse events. For example, studies typically exclude individuals with a history of an eating disorder, history of bariatric surgery, active suicidal ideation, major depression or other significant mental illness, severe gastrointestinal disease, and a history of pancreatitis [2,3]. Thus, data are lacking about drug safety and efficacy when used by such individuals in the general population. Drug–drug interactions also must be carefully considered. For example, delayed gastric emptying can reduce absorption of oral contraceptives, potentially diminishing their effectiveness, while concomitant use of GLP-1 RAs with insulin or sulfonylureas increases the risk of hypoglycemia [6]. These interactions underscore the need for careful clinical oversight rather than casual prescribing. The rapid expansion of GLP-1 prescribing has outpaced the evidence base in some areas. Clinicians and patients must weigh the potential benefits and risks of GLP-1s, highlighting the importance of individualized prescribing, close monitoring, and shared decision-making.

Post-marketing surveillance, real-world evidence, and case reports remain essential for identifying potential risks that only emerge when these agents are used in broader and more diverse populations. Beyond these risks, ongoing clinical and scientific discussions highlight the potential for loss of lean muscle mass and bone density with significant weight reduction, both of which could have important implications for long-term health outcomes, particularly in older adults [7]. Micronutrient deficiencies also remain a concern and may necessitate monitoring and supplementation [8]. Ultimately, the long-term effectiveness of GLP-1s outside controlled trials remains unclear. Questions persist around sustained weight loss, cardiometabolic outcomes, and discontinuation effects, all of which require longitudinal, real-world studies. Building these evidence bases through registries, pragmatic trials, and post-marketing surveillance will be critical to inform guidelines, optimize care, and balance innovation with safety.

## When supply cannot meet demand: Health system responses to GLP-1 popularity

The rapid uptake of GLP1 RAs has highlighted several health system-level challenges, such as limited provider knowledge and capacity for obesity treatment, access concerns, and monitoring and follow-up gaps [9]. This underscores the urgent need for deliberate system-level planning, as well as enhanced infrastructure and coordination to manage these changes effectively.

Spurred by widespread pharmacy shortages of semaglutide in March 2022 and tirzepatide in 2023, as well as high prices of GLP-1-based medications, many individuals sought alternatives, leading to an increase in the compounding of GLP-1 medications. While shortages have since resolved—and the US Food and Drug Administration (FDA) has issued guidance cautioning against compounding when FDA-approved products are available—shortages created a gray area where pharmacies and compounding facilities stepped in to fill unmet needs (and may inappropriately continue to do so). Compounding introduces significant concerns, including variability in quality, dosing accuracy, stability, and potential safety risks [10]. Reports of inconsistencies in compounded semaglutide and tirzepatide highlight the challenges in ensuring therapeutic equivalence to the branded formulations. The tension between patient access and regulatory oversight underscores the importance of robust supply chains, transparent manufacturing practices, and ongoing monitoring of compounded alternatives.

Predatory marketing practices have led to the proliferation of unregulated products such as so-called “GLP-1 patches” and unvalidated “microdosing” regimens [10]. These approaches are not supported by pharmacokinetic or pharmacodynamic evidence. GLP-1 RAs are peptide molecules that cannot yet be effectively absorbed through transdermal delivery systems, and microdosing has not been demonstrated to achieve therapeutic plasma concentrations. Such products exploit public demand for weight loss therapies, posing risks of treatment failure, adverse effects from unknown or adulterated ingredients, and delays in accessing evidence-based care. Clear communication of the scientific limitations of these practices, coupled with regulatory oversight, is necessary to protect patients and ensure the safe, effective use of GLP-1 therapies.

The expansion of GLP-1-based therapies has been driven not only by clinical adoption but also by the rapid rise of direct-to-consumer (DTC) platforms. Although DTC models have long been present in obesity care, new and expanding companies have capitalized on unprecedented public demand for incretin-based therapies, positioning themselves as alternatives to traditional healthcare encounters. By offering quick online questionnaires, telehealth consultations, and direct-to-home delivery of medications, DTC platforms reduce logistical barriers such as wait times, travel, and insurance hurdles. For some patients, this model may feel more approachable, efficient, and affordable. In this way, DTC companies have helped normalize GLP-1 therapies in the broader cultural conversation, moving them from specialized clinical tools into mainstream awareness, further fueling widespread adoption.

However, the DTC model also raises substantial concerns about patient safety, care quality, and long-term outcomes. Because many platforms operate with minimal clinical screening, important contraindications may go undetected. Additionally, patients may not be adequately prepared for common side effects such as nausea, constipation, or vomiting, nor for more serious risks that require medical follow-up. Fragmentation of care further compounds these issues; when patients do not disclose DTC prescriptions to their primary care providers, it becomes more difficult to coordinate treatment plans and monitor for drug–drug interactions, duplication of therapy, or progression of comorbid conditions like diabetes, hypertension, or dyslipidemia. Preventive care opportunities may also be lost if patients substitute DTC prescribing for ongoing clinical relationships. In some cases, reliance on DTC channels may even delay the recognition of underlying medical conditions that contribute to weight gain, such as endocrine disorders or mental health challenges, leaving patients without comprehensive treatment.

To balance accessibility with safety, clear regulatory standards, stronger clinical oversight, and better integration between DTC providers and traditional healthcare systems are needed. Ultimately, the challenge lies in ensuring that the rapid expansion of DTC prescribing for GLP-1 therapies strengthens, rather than undermines, the broader goals of safe, effective, and patient-centered obesity care.

## Conclusions

The rapid adoption of GLP-1-based medications represents a paradigm shift in obesity treatment and in healthcare more broadly, offering efficacy for some patients that approaches that of surgical interventions. Yet without careful attention to long-term safety and integration into comprehensive care models, the promise of these agents risks being undermined by inappropriate use and potential safety lapses. The challenge for clinicians, policymakers, and industry stakeholders is to balance innovation with caution, ensuring that these powerful medications are used in a manner that maximizes benefit, minimizes harm, and aligns with the broader goals of public health.

## Abbreviations

DTC: direct-to-consumer
FDA: Food and Drug Administration
GLP-1: glucagon-like peptide-1
RA: receptor agonist

## References

[1] Mahase E. GLP-1 agonists: US sees 700% increase over four years in number of patients without diabetes starting treatment. BMJ. 2024. 10.1136/bmj.q1645 39043396

[2] Jastreboff AM, Aronne LJ, Ahmad NN, Wharton S, Connery L, Alves B, et al. Tirzepatide once weekly for the treatment of obesity. N Engl J Med. 2022;387(3):205–16. doi: 10.1056/NEJMoa2206038 35658024

[3] Wilding JPH, Batterham RL, Calanna S, Davies M, Van Gaal LF, Lingvay I, et al. Once-weekly semaglutide in adults with overweight or obesity. N Engl J Med. 2021;384(11):989–1002. doi: 10.1056/NEJMoa2032183 33567185

[4] Ismaiel A, Scarlata GGM, Boitos I, Leucuta D-C, Popa S-L, Al Srouji N, et al. Gastrointestinal adverse events associated with GLP-1 RA in non-diabetic patients with overweight or obesity: a systematic review and network meta-analysis. Int J Obes (Lond). 2025;49(10):1946–57. doi: 10.1038/s41366-025-01859-6 40804463 PMC12532569

[5] O’Dowd A. GLP-1 receptor agonists: MHRA to study possible side effects after acute pancreatitis cases. BMJ. 2025;389:r1344. doi: 10.1136/bmj.r1344 40578853

[6] Min JS, Jo SJ, Lee S, Kim DY, Kim DH, Lee CB, et al. A comprehensive review on the pharmacokinetics and drug-drug interactions of approved GLP-1 receptor agonists and a dual GLP-1/GIP receptor agonist. Drug Des Devel Ther. 2025;19:3509–37. doi: 10.2147/DDDT.S506957 40330819 PMC12052016

[7] Linge J, Birkenfeld AL, Neeland IJ. Muscle mass and glucagon-like peptide-1 receptor agonists: adaptive or maladaptive response to weight loss?. Circulation. 2024;150(16):1288–98. doi: 10.1161/CIRCULATIONAHA.124.067676 39401279

[8] Scott Butsch W, Sulo S, Chang AT, Kim JA, Kerr KW, Williams DR, et al. Nutritional deficiencies and muscle loss in adults with type 2 diabetes using GLP-1 receptor agonists: a retrospective observational study. Obes Pillars. 2025;15:100186. doi: 10.1016/j.obpill.2025.100186 40584822 PMC12205620

[9] Pearson SD, Whaley CM, Emond SK. Affordable access to GLP-1 obesity medications: strategies to guide market action and policy solutions in the US. J Comp Eff Res. 2025;14(9):e250083. doi: 10.57264/cer-2025-0083 40641455 PMC12403326

[10] McCall KL, Mastro Dwyer KA, Casey RT, Samana TN, Sulicz EK, Tso SY, et al. Safety analysis of compounded GLP-1 receptor agonists: a pharmacovigilance study using the FDA adverse event reporting system. Expert Opin Drug Saf. 2025;:1–8. doi: 10.1080/14740338.2025.2499670 40285721

